# Retraction: Construction of a binary S-scheme S-g-C_3_N_4_/Co-ZF heterojunction with enhanced spatial charge separation for sunlight-driven photocatalytic performance

**DOI:** 10.1039/d6ra90075e

**Published:** 2026-07-22

**Authors:** Ali Bahadur, Shahid Iqbal, Mohsin Javed, Syeda Saba Hassan, Sohail Nadeem, Ali Akbar, Rami M. Alzhrani, Murefah Mana Al-Anazy, Eslam B. Elkaeed, Nasser S. Awwad, Hala A. Ibrahium, Ayesha Mohyuddin

**Affiliations:** a Department of Chemistry, College of Science and Technology, Wenzhou-Kean University Wenzhou China; b Department of Chemistry, School of Natural Sciences (SNS), National University of Science and Technology (NUST) H-12 Islamabad 46000 Pakistan shahidiqbal.chem@sns.nust.edu.pk; c Department of Chemistry, School of Science, University of Management and Technology Lahore Pakistan; d Department of Physics, University of Agriculture Faisalabad (UAF) Faisalabad Punjab 38000 Pakistan; e Department of Pharmaceutics and Industrial Pharmacy, College of Pharmacy, Taif University P. O. Box 11099 Taif 21944 Saudi Arabia; f Department of Chemistry, College of Science, Princess Nourah bint Abdulrahman University P. O. Box 84428 Riyadh 11671 Saudi Arabia; g Department of Pharmaceutical Sciences, College of Pharmacy, AlMaarefa University Riyadh 13713 Saudi Arabia; h Chemistry Department, Faculty of Science, King Khalid University P. O. Box 9004 Abha 61413 Saudi Arabia; i Biology Department, Faculty of Science, King Khalid University P. O. Box 9004 Abha 61413 Saudi Arabia; j Department of Semi Pilot Plant, Nuclear Materials Authority P. O. Box 530 El Maadi Egypt

## Abstract

Retraction of “Construction of a binary S-scheme S-g-C_3_N_4_/Co-ZF heterojunction with enhanced spatial charge separation for sunlight-driven photocatalytic performance” by Ali Bahadur *et al.*, *RSC Adv.*, 2022, **12**, 23263–23273, https://doi.org/10.1039/D1RA08525E.

The Royal Society of Chemistry hereby wholly retracts this *RSC Advances* article due to concerns with the reliability of the data.

The XRD patterns for ZF, 6% Co-ZF and 48% S-g-C_3_N_4_/6% Co-ZF in Fig. 1 are partially identical in the region 11–22 2*θ*.

The degradation experiments in Fig. 4a–d and transient photocurrent results in Fig. 5b were first published in ref. 1 and described as different experiments.

The XPS data in Fig. S1f and the ESR data in S2a were first published in ref. 2 and described as different experiments.

The authors provided a response and new data but they did not address the concerns and they were not able to provide the original raw data.

Given the significance of these concerns, the findings presented in this paper are no longer reliable.

Ali Akbar and Shahid Iqbal oppose this retraction decision. The other authors did not respond to any correspondence regarding the retraction of this article. Rami M. Alzhrani, Nasser S. Awwad and Sohail Nadeem could not be contacted.

This retraction supersedes the information provided in the Expression of Concern related to this article.

Signed: Laura Fisher, Executive Editor, *RSC Advances*

Date: 16th July 2026

## References

[cit1] Bahadur A., Iqbal S., Alsaab H. O., Awwad N. S., Ibrahium H. A. (2021). RSC Adv..

[cit2] Abubshait H. A., Iqbal S., Abubshait S. A., Alotaibi M. T., Alwadai N., Alfryyan N., Alsaab H. O., Awwad N. S., Ibrahium H. A. (2022). RSC Adv..

